# Treatment of Palatal Myoclonus with Botulinum Toxin Injection

**DOI:** 10.1155/2013/231505

**Published:** 2013-10-08

**Authors:** Mursalin M. Anis, Natasha Pollak

**Affiliations:** Department of Otolaryngology-Head & Neck Surgery, Temple University School of Medicine, 3440 North Broad Street, Kresge West, Philadelphia, PA 19140, USA

## Abstract

Palatal myoclonus is a rare cause of pulsatile tinnitus in patients presenting to the otolaryngology office. Rhythmic involuntary contractions of the palatal muscles produce the pulsatile tinnitus in these patients. Treatment of this benign but distressing condition with anxiolytics, anticonvulsants, and surgery has been largely unsuccessful. A few investigators have obtained promising results with botulinum toxin injection into the palatal muscles. We present a patient with palatal myoclonus who failed conservative treatment with anxiolytics. Unilateral injection of botulinum toxin into her tensor veli palatini muscle under electromyographic guidance resolved pulsatile tinnitus in her ipsilateral ear and unmasked pulsatile tinnitus in the contralateral ear. A novel method of following transient postinjection symptoms using a diary is presented in this study. Botulinum toxin dose must be titrated to achieve optimal results in each individual patient, analogous to titrations done for spasmodic dysphonia. Knowledge of the temporal onset of postinjection side effects and symptomatic relief may aid physicians in dose titration and surveillance. We present suggestions on titrating the botulinum toxin dose to optimal levels. A review of the literature on the use of botulinum toxin for palatal myoclonus and some common complications are discussed.

## 1. Introduction

Palatal myoclonus is a rare cause of pulsatile tinnitus in patients presenting to the otolaryngology clinic [1]. Rhythmic involuntary contraction of palatal muscles produces the pulsatile tinnitus in these patients. Two variants are described in the literature, a “symptomatic form” of palatal myoclonus that is due to brainstem or cerebellar lesions and an “essential form” that occurs in isolation and has no known intracranial pathology associated with it [2]. 

 Investigational workup for palatal myoclonus involves audiometric studies to delineate middle and inner ear diseases along with magnetic resonance imaging of the brain. Previous treatments of this benign but distressing condition with anxiolytics, anticonvulsants, and surgery have been largely unsuccessful. White noise masking has also been tried and has resulted in modest symptomatic relief [3]. 

Few investigators have described the use of botulinum toxin injection into the palatal muscles to treat palatal myoclonus [2, 3]. Botulinum toxin is injected into the tensor veli palatini and/or the levator veli palatini muscles. Clostridium botulinum toxin is a protein that inhibits release of acetylcholine from the presynaptic nerve terminals, thereby essentially causing local “chemical denervation.” Accidental systemic injection is well documented and is fatal. The effect of botulinum toxin persists from 2 to 28 days leading to weakness and atrophy of the injected muscles [3]. 

There have been few small case series documenting the success and side effects of injecting botulinum toxin for treatment of palatal myoclonus. Some side effects include hypernasality, velopharyngeal insufficiency (VPI) leading to nasopharyngeal regurgitation, dysphagia, and need for repeat injections. Side effects are minimized with the use of electromyographic guidance to inject the area with highest myoclonic activity and optimizing the injected toxin dose to obtain desirable effects with minimal or no toxicity. Analogous to botulinum toxin treatment of spasmodic dysphonia, optimal dose must be determined for each individual patient.

## 2. Case Description and Methods

A 36-year-old woman complained of a noise similar to “bubbling water” and “popping bubble wraps” in her left ear continuously for the past year. She described the pulsatile tinnitus asynchronous with her pulse. She was distressed by lack of sleep due to the constant noise. She denied any hearing loss, headaches, vertigo, otalgia, or otorrhea. She had no history of acoustic trauma, prior otologic surgeries, or otitis media. She had not tried any treatment to alleviate her tinnitus. She took loratadine for her seasonal allergies. She had no family history of otologic diseases. She is a homemaker who smoked water pipe occasionally but denied any alcohol intake. 

Her physical examination was notable for a normal otoscopic exam, including normal pneumatic otoscopy. Examination of her oral cavity revealed rhythmic contraction of both sides of her soft palate. Contractions were asynchronous with patient's pulse. No neck masses or carotid bruits could be auscultated. An audiogram showed normal hearing and normal tympanograms bilaterally. An MRI of the brain was normal (not shown). Patient was diagnosed with essential palatal myoclonus. She was initially prescribed diazepam but experienced no relief of tinnitus. Patient was then informed of the botulinum toxin injection option for treatment of palatal myoclonus. The following risks were identified in the literature and were explained to the patient: dysphagia, velopharyngeal insufficiency with hypernasal speech, aural fullness, local irritation/inflammation of injection site, need for further procedures such as Botox dose titration, Eustachian tube dysfunction with need for tympanostomy tube placement, and inherent transient benefit of botulinum toxin injection [2, 3]. 

**Figure 1:**
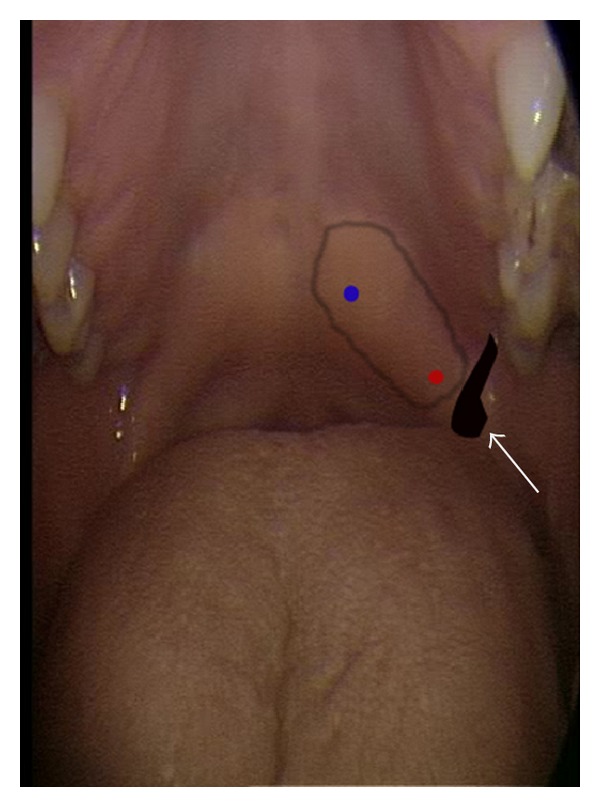
Injection site of botulinum toxin into insertion and aponeurosis of tensor veli palatini. Optimum relief of pulsatile tinnitus obtained with 7.5 units of botulinum toxin injected (red dot) just medial to pterygoid hamulus (white arrow) and 3 units of botulinum toxin injected just lateral to musculus uvulae (blue dot).

Patient was brought to the ENT procedure room. Bilateral palatal myoclonus was documented videographically. Under electromyographic guidance, a total of 10.5 U of botulinum toxin type A was injected in the tensor veli palatini muscle without any local anesthetic. Specifically, 7.5 U was injected into the lateral soft palate just medial to the pterygoid hamulus and 3 U into the medial soft palate just lateral to musculus uvulae (Figure 1). Patient tolerated the procedure well.

Patient returned 5 days after injection with complete relief of her left-sided pulsatile tinnitus. She noted some mild dysphagia that developed 2 days after injection but resolved within a week. Also, minimal pulsatile tinnitus in her right ear was now unmasked.

On examination, she had symmetric palatal elevation and no palatal twitching. She was satisfied with the procedure. She achieved symptomatic relief for about 3–5 months. Over the course of 2 years, she subsequently received 3 more injections of botulinum toxin into her left tensor veli palatini and one injection into her right tensor veli palatini due to unmasking of milder tinnitus on the right. 

She kept a diary of her symptoms after injection. Using the diary, the patient tracked the following parameters for 14 days and rated them on a scale from 0–10: pulsatile tinnitus, dysphagia, voice changes, ear pressure, clicking in contralateral ear, and velopharyngeal insufficiency which was described to the patient as liquid escaping into the nose upon swallowing. 

**Figure 2:**
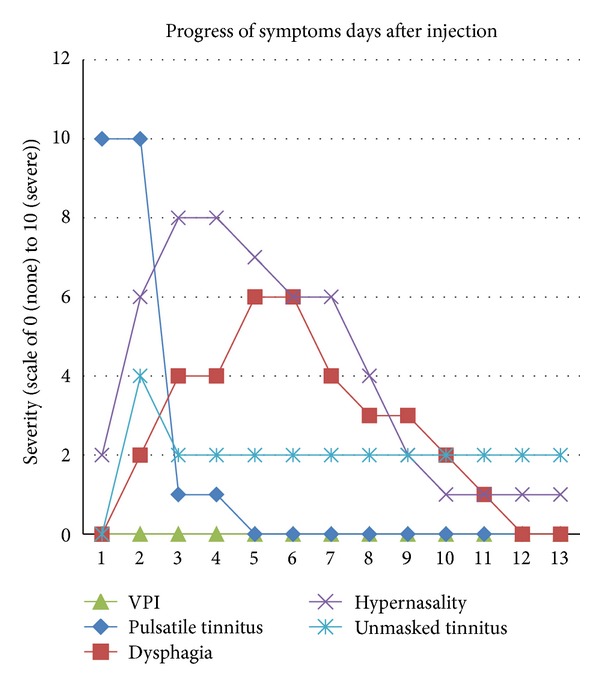
Progress of symptoms days after injection. Temporal onset and duration of symptoms over the course of 2 weeks after injection of 10.5 units of botulinum toxin into left tensor veli palatini. Temporal relationship of side effects of velopharyngeal insufficiency (VPI), dysphagia, hypernasality, and unmasking of tinnitus in contralateral ear is compared with symptomatic relief of pulsatile tinnitus in left ear (blue diamond).

Patient's diary of symptoms after injections was used to titrate the dose of botulinum toxin to the desired effect while minimizing duration and severity of side effects. For our patient, a dose of 10.5 U was found to achieve symptomatic relief within 2 days of injection and limit duration of side effects to 1 week (Figure 2). 

## 3. Discussion

Palatal myoclonus (PM) is classified either as a symptomatic or essential tremor. Symptomatic PM is due to brainstem or cerebellar lesions in the triangle of Guillain-Mollaret involving the dentato-rubro-olivary pathway and affecting most often the levator veli palatine muscle [4, 5]. The associated hypertrophic olivary degeneration has a specific appearance on MRI, namely, high T2 signal in the area of the inferior olives [6]. Essential PM has been described as either idiopathic, psychogenic, voluntary, central, or peripheral in origin without any focal neurological lesions on imaging [7]. Essential PM affects mostly the tensor veli palatini muscle and has been controlled successfully with transient chemical denervation using botulinum toxin A. Our patient received botulinum toxin injection to her left tensor veli palatini to address her left-sided pulsatile tinnitus. Her right-sided pulsatile tinnitus responded to a lower dose of 7.5 units of botulinum toxin and was mildly unmasked but did not appear to bother her as she obtained complete relief from her left-sided tinnitus.

As others have noted, botulinum toxin injection for palatal myoclonus is a minimally invasive, safe treatment option that provides reproducible benefit. Risks of dysphagia, velopharyngeal insufficiency, hypernasal speech, aural fullness, local irritation of injection site, transient benefit of botulinum toxin injection, and possibly unmasking contralateral tinnitus should be discussed with patients. In this case, onset of tinnitus relief occurs between days 2 and 3 after Botox injection. Dysphagia reached moderate levels at day 5-6 after injection and resolved within 2 weeks. Hypernasal speech peaked at day 2-3 after injection and also resolves in less than 2 weeks. Velopharyngeal insufficiency and aural fullness did not occur during Botox treatment. Titrating the Botox dose on subsequent injections allowed us to minimize both severity and duration of side effects. As the optimal dose and location of injections for this patient are now known, follow-up injections can be done faster, without electromyographic guidance, and with minimal discomfort and minimal interruption of her daily routine. Satisfactory results are attainable with cooperation of the patient, based on understanding of possible risks and need for repeat injections to maintain benefit. 

## References

[1] Sismanis A (2003). Pulsatile tinnitus. *Otolaryngologic Clinics of North America*.

[2] Penney SE, Bruce IA, Saeed SR (2006). Botulinum toxin is effective and safe for palatal tremor: a report of five cases and a review of the literature. *Journal of Neurology*.

[3] Saeed SR, Brookes GB (1993). The use of clostridium botulinum toxin in palatal myoclonus: a preliminary report. *Journal of Laryngology and Otology*.

[4] Seidman MD, Arenberg JG, Shirwany NA (1999). Palatal myoclonus as a cause of objective tinnitus: a report of six cases and a review of the literature. *Ear, Nose and Throat Journal*.

[5] Deuschl G, Toro C, Valls-Sole J, Zeffiro T, Zee DS, Hallett M (1994). Symptomatic and essential palatal tremor: 1. Clinical, physiological and MRI analysis. *Brain*.

[6] Goyal M, Versnick E, Tuite P (2000). Hypertrophic olivary degeneration: metaanalysis of the temporal evolution of MR findings. *American Journal of Neuroradiology*.

[7] Zadikoff C, Lang AE, Klein C (2006). The ‘essentials’ of essential palatal tremor: a reappraisal of the nosology. *Brain*.

